# Predictive voting model for early diagnosis of diabetes dataset

**DOI:** 10.3389/fcvm.2026.1780009

**Published:** 2026-05-28

**Authors:** Talaa Al-Bimani, Jamil AlShaqsi, Rami Alkhawaldeh, Adil S. Al Busaidi, Ali Tarhini

**Affiliations:** 1 Information Systems Department, Sultan Qaboos University, Muscat, Oman; 2 Computer Information Systems Department, The University of Jordan, Aqaba, Jordan; 3 Innovation and Technology Transfer Center, Sultan Qaboos University, Muscat, Oman

**Keywords:** classification, data mining, diabetes in Oman, knowledge discovery in database (KDD), prediction algorithms, voting ensemble algorithms

## Abstract

This research aimed to construct predictive voting models (hard vote and soft vote) to improve the diabetes diagnosis system at the initial pre-diabetes stage using several risk factors retrieved from a dataset collected from a government hospital in Oman. The study focused on identifying significant predictors of diabetes and enhancing the accuracy of early diagnosis. The Knowledge Discovery in Database (KDD) model was utilized to conduct the experiments. A 33-month historical dataset comprising *N* = 4104 registered patients and 14 variables was analyzed. The features used for diabetes classification included age, height, weight, gender, diastolic and systolic blood pressure, cholesterol level, blood glucose level, and haemoglobin level. Five supervised classification algorithms were applied to construct the voting models: Decision Tree (J48), K-Nearest Neighbors (KNN), Support Vector Machine (SVM), Random Forest, and Naïve Bayes. The findings revealed that the hard-vote model achieved the highest predictive accuracy of 84.7% compared with the soft-vote model. Additionally, the haemoglobin A1C test (HbA1c), Fasting Plasma Glucose (FPG), and age were identified as the most significant factors for predicting diabetes. The extracted rules indicated that HbA1c served as the initial criterion for diabetes diagnosis, with a threshold value of 6.3. The study demonstrated the effectiveness of ensemble voting models in improving diabetes prediction during the pre-diabetes stage. The identified predictors and extracted rules may support healthcare professionals in making earlier and more accurate diagnostic decisions. Furthermore, the involvement of domain experts and the validation of rules using classified patient cases strengthened the reliability and practical applicability of the proposed models.

## 1 Introduction

**Figure 1:**
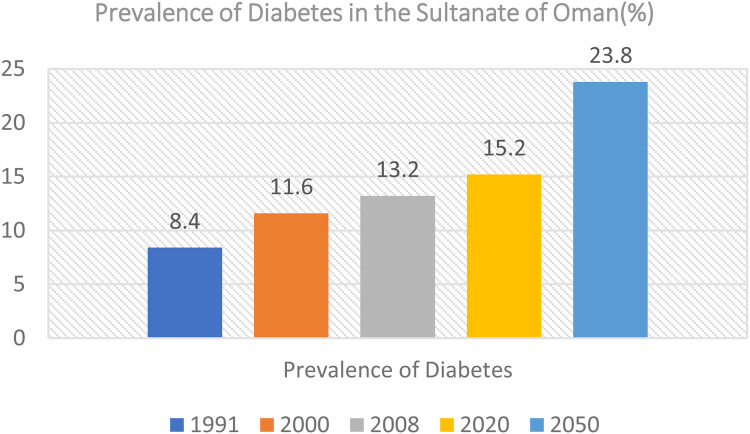
The prevalence of diabetes in the sultanate of Oman from 1991 to 2020 and the projection of 2050.

Diabetes Mellitus (DM) is a severe and chronic disease defined as an abnormal metabolic disease characterised by increased blood sugar resulting from either lack of insulin secretion or ineffective insulin function (1–3). Worldwide,10% of total diabetes develop type 1 diabetes, and approximately 90% of total patients develop type Ⅱ diabetes (4). In the Sultanate of Oman, the prevalence of diabetes has increased as a significant health dilemma due to the high rate of pre-diabetes, overweight, and obesity (5). Figure 1 shows the prevalence of diabetes in the Sultanate of Oman from 1991 to 2020 and the projection up to 2050. The first National Diabetes Survey conducted in 1991 by the World Health Organisation (WHO), which comprises both males and females, shows that the prevalence of DM aged 20 and above among Omanis was 8.4% (6). A comparable National health survey conducted in 2000 indicated that DM prevalence increased to 11.6% and headed to 13.2% in the subsequent study performed in 2008. The prevalence of diabetes is also expected to grow over the next two decades, from 15.2% in 2020 to 23.8% in 2050 (7, 66).

The global burden of disease report shows that the epidemiological transition of leading diseases causes health loss in Oman, and diabetes has moved from the 12th rank in 1990 to the 2nd in 2010 (8). However, the current screening system used to diagnose diabetes patients is extremely limited to a specific range of people. The existing screening system is categorised into three situations (6). The first one is when the patient presents typical symptoms, and the second screening is for individuals aged forty and above, conducted via a noncommunicable diseases (NCDs) program organised once every three years. The last is opportunistic screening for high-risk individuals. Hence, lack of routine screening at a younger age and late diagnosis of diabetes resulted in higher morbidity and increased treatment costs in the county (9). Prevention and delay from developing diabetes occur at the initial stage of diagnosis and simple lifestyle changes and diet (9). Therefore, effective diabetes screening should be implemented to enhance the quality of life and minimise the cost of the health care system (10).

Data mining is a substantial process defined as the process of extracting previously unknown but potentially useful information from a given dataset (11). Traditionally the diagnosis depends on the doctor's experience that might produce inaccurate results (12). Hence, employing data mining techniques particularly predictive models in the hospitals can accurately diagnose diabetes at the earliest stage of the pre-diabetes (13) by utilising the historical data of the patient available in the system. Information overload in healthcare centres (14) needs a sophisticated tool to analyse and provide a report for planning and decision-making. since manual data analysis is highly subjective and time-consuming and has become impractical for healthcare (15). Thus, data mining techniques are required to extract exciting patterns hidden in massive amounts of the dataset (16, 17). Significantly, Data mining has become an active research tool in healthcare (18, 19) to analyse biological data and solve biological problems.

Therefore, this research aims to apply data mining techniques to construct the predictive ensemble models for improving the diabetes diagnosis system that can predict diabetes at the initial pre-diabetes stage using several patient-related factors retrieved from a government hospital dataset. The detailed objective of this research is as follows:

1. To construct predictive ensemble voting models using the most applied five supervised classification algorithms in predicting diabetes and evaluate their performance to identify the most optimal ensemble model based on the highest predictive accuracy.
2. To use the outperformed ensemble model to demonstrate the exploitation of the model for the clinical setting using a new dataset that consists of the unknown target variable.
3. To apply a classification algorithm to extract valuable knowledge available in the dataset collected from the SQUH.

The remaining parts of the paper contain eight sections. Section 2 describes the related works. Section 3 explains different classification algorithms that have been applied to conduct experiments. Section four consists of the research methodology based on the KDD process model. Section five illustrates the experimental results and the discussion. Section six includes the implications of the research. The conclusion was included in section seven. The limitations and future work were summarised in the last section.

## 2 Related work

The Diabetes Mellitus (DM) screening studies were conducted in the Sultanate of Oman using different methodologies. The descriptive and predictive methods were applied to identify the individual at considerable risk of developing diabetes by creating diabetic risk scores rather than classifying the incidence of either TRUE or FALSE diabetes.

In the study conducted by Al-Lawati and Tuomilehto (20), a risk score for self-assessment was created to identify individuals at considerable risk of developing type Ⅱ diabetes in the Omani community. Two cohorts were applied. The first dataset was collected from the National Diabetes Survey conducted in 1991 with 4,881 instances to build the Logistic Regression model. Another dataset collected from the Nizwa survey conducted in 2001 consisted of five similar attributes with 1,432 instances applied to assess the model's validity.

Barakat et al. (21) proposed a hybrid model that includes K-means for clustering and SVM for classification. The dataset collected during the National Survey conducted in 1991 consists of 4,682 instances with 10 attributes. Different methods were applied to extract rules from the dataset, including DT, SQRex-SVM, and Eclectic SVM methods. The study found that fasting blood sugar, waist circumference, and diastolic blood pressure appeared as significant antecedent factors for all used algorithms. The prediction results obtained were 94% accuracy, sensitivity 93%, and specificity 94%. However, the study calls for future work to develop a more sophisticated tool in the prediction of diabetes to minimise healthcare costs through early prediction of diabetes.

D'Souza et al. (22) researched to evaluate the risk assessment scoring system for the initial screening of diabetes by selecting Omani adults in Muscat. The “Finnish Diabetes Risk Score (FINDRISKC)” predictive method was used to collect data in 2009 by randomly selecting ninety-three (93) samples of the Omani population. According to this designed questionnaire, the risk assessment included six attributes. The data were analysed using statistical methods (SPSS) to provide a descriptive summary of the risk assessment scoring values that predict the development of diabetes type 2 using the Pearson chi-square test.

Al-Lawati et al. (23) proposed the logistic regression model to predict the diabetes risk score using the dataset collected from two cross-sectional surveys. The dataset (instances = 2,720) was collected during a national survey in 2008 by the World Health Survey and was used to build the predictive model; the dataset (instances = 1,355) collected in 2006 using Sur Survey was applied for the model validation with similar 11 attributes.

The above studies applied traditional statistical tools to analyse the statistical dataset. However, minimal research used data mining techniques to predict and classify diabetes in the Sultanate of Oman. Besides, none of the studies were conducted to apply the historical patients' dataset in the SQUH database. Accordingly, there is a practical need to conduct studies in the Sultanate of Oman to apply sophisticated data mining techniques to improve the diabetic screening system using the hospital's historical patient dataset.

According to international researchers, several studies have been conducted to predict diabetes using individual and ensemble data mining techniques.

The study conducted by Saritas and Yasar (24) in predicting breast cancer using Artificial Neural Network (ANN) and Naïve Bayes (NB). The results prove that NB algorithms achieved the ANN by being accurate at 86.95% in classifying the patients compared to ANN, which has 83.54%. However, in predicting diabetes using the Pima Indian Diateds Dataset (PIDD) dataset, the NB algorithm has an accuracy of 76.33% (12).

Hassan et al. (25) also apply three Classification techniques including Decision Tree (DT), K-Nearest Neighbour (KNN)and Support Vector Machine(SVM) predicting diabetes and non-diabetes using the (PIDD) dataset. The dataset consists of 768 instant with 9 attributes including Pregnancy, glucose, insulin, blood pressure, skin thickness, BMI, pedigree, Age and class diabetes and non-diabetes. The Results prove the SVM outperforms the other algorithms with 90.23% accuracy compared to KNN and DT with 75.97% and 75.32% respectively.

A study by Kazerouni et al. (26) examined which classification algorithm offered the best prediction results for diabetes based on a subset of genetic factors (long non-coding RNA). The dataset was collected from the previous studies conducted in Iranian hospitals. It consists of 200 instances with 100 healthy people and 100 diabetes type Ⅱ. The proposed algorithms are KNN, SVM logistic regression (LR), DT, (ANN) applied in ANACONDA3–5.2.0 64-bit. Ten-fold stratified cross-validation. The study found that it is impossible to conclude that one classification technique always works best. SVM and logistic regression outperformed the other algorithms by 95% in the AUC curve. While KNN achieves the highest sensitivity of 96%, the highest specificity belongs to SVM at 86%. ANN also attained a high AUC curve with a minimum Standard deviation of 93% and SD 0.03, respectively, compared to other algorithms.

Furthermore, the manuscript conducted by Sathar et al. (27) focuses on the comparative analysis of algorithms to enhance the prediction model's accuracy using data mining techniques including SVM, NB, KNN, DT, (LR), and Random Forest (RF) using the PIDD dataset. The results obtained were SVM has 78% accuracy while LR, KNN, NB and DT have 77%, 75%, 74% and 69% respectively. The study can be enhanced by applying a large dataset with similar algorithms.

Regarding Ensemble voting models, Prema et al. (28) proposed a voting classifier with several individual algorithms, including KNN, NB, DT, LR Linear SVM, RF, RBF SVM, Gaussian Proc, AdaBoost, and QDA ensembled. They aggregated the prediction results using the majority strategy to generate the final prediction Using the PIDD dataset. Two experiments were conducted using PIMA; the first data set was divided into 30% and 70% for testing and training, respectively, and the second used 10-fold cross-validation. The prediction results achieved were 80.95% and 79.53% accuracy in both experiments.

A recent study by Kumari et al. (13) proposed a soft voting classifier that combines three machine learning algorithms, Logistic Regression (LG), Naive Bayes (NB), and Random Forest (RF) to generate an ensemble model for diabetes classification using PIDD dataset. The final prediction results show that the proposed algorithm achieved 79.04% accuracy, while the precision, recall, and F1 score values with 73.48%, 71.45%, and 80.6%, respectively. The proposed model has been applied to another breast cancer dataset to validate its robustness, and the results obtained were higher than the diabetes dataset.

From the above information we can conclude that previous diabetes prediction studies exhibit several common limitations that motivate the present work. Many earlier studies in Oman primarily employed traditional statistical risk scoring and logistic regression techniques instead of more advanced data mining and ensemble learning approaches, and were largely based on survey or questionnaire data rather than real-world hospital electronic records. Likewise, numerous international machine-learning studies relied on small benchmark datasets, such as PIDD, which constrains generalizability and practical clinical applicability. Research on voting ensemble models remains limited, and existing implementations typically used a narrow set of base classifiers and reported modest performance gains. Moreover, much of the prior literature focused mainly on reporting accuracy results without demonstrating real clinical deployment, validation on unseen operational data, or extraction of interpretable rules for clinician support (29). Limited attention has also been given to class imbalance treatment, comprehensive preprocessing of noisy medical data, and statistical significance testing of improvements over single models. Together, these limitations indicate a clear need for a robust, clinically applicable, ensemble-driven, and statistically validated prediction framework built on real hospital datasets (4, 21, 30).

## 3 Classification algorithms

Classification is an attempt to predict the condition of a categorical variable (11). Supervised classification aims to construct the model of class label distributions based on their features (10). Then the classifier is used to predict new data into the predefined categorized classes (31, 32). Numerous machine-learning algorithms have been proposed and used in a variety of medical applications (33–35). However, it is an essential part of data mining to select the appropriate algorithm, which depends on the classification algorithm's requirement outperforming the other (36). Furthermore, to improve the predictive accuracy due to the combination of different decision opinions from diverse base classifiers to minimise variance over a single classifier (13, 36–38). Therefore, this study applied SVM, DT(J48), RF, KNN, and NB to build voting ensemble models as the most implemented algorithms in the prediction and classification of diabetes based on recent studies. These are as follows:

Support Vector Machine (SVM): classify the categorical variable by identifying the best hyperplane and segregating the data into classes (17). It draws the border separator's hyperplane (decision boundary) using the nearest data point called support vectors (39). The hyperplane is tuned to optimise its distance from the decision boundary to correctly classify the data (40). SVM consists of a hard margin, separating the data using a linear separable line. A soft margin separates the non-linear data by allowing the algorithm to make mistakes while maximizing the margin distance with minimum error. SVM has been used for several applications in different research fields, such as feature selection (41), drug discovery, identification of cancer (42), and early detection of diabetes (25–27, 43).

K- Nearest Neighbours (KNN): is a lazy learner classifier because it holds all available cases and calculates the distance between the nearest data points using Euclidean distance to classify the new instances based on the majority votes of the nearest neighbours (27, 32, 44). Thus, it is effective in various real-world domains, such as the classification and prediction of diabetes (25, 45).

Decision Tree (DT): is the graphical representation of the features made based on the information gained from each attribute given by the target classes. The highest information gained attribute is selected as a root node (46) and used to split the tree according to its instances to generate the second level of the tree. The process is recursive until it reaches the leaf node representing the target class throughout the internal node. The study applies the J48 algorithm, the extension of the decision tree C4.5 classifier developed by the WEKA project team for classification (67). DT provides a comprehensive description of how the instances belong to a specific class (32). It is also recommended by (47) as the best feature selection algorithm. The decision tree has been used in different data mining applications in bioinformatics (46). For example, it generates rules for intrusion detection and diagnosis of different diseases such as diabetes, cardiovascular disease, and cancers.

Naïve Bayes (NB): this is the most common probabilistic algorithm that applies conditional probability to predict and classify the instances by counting the occurrence of a given feature based on the output values in a dataset (24). It is the most straightforward implementing algorithm of the Bayesian theory, assuming that all attributes are independent of each other, considering the class variable's value (12). Also, it is computationally fast and operates in a small data set for training and selecting parameters for the classification (48). This algorithm has been widely used to diagnose chronic diseases like breast cancer and diabetes (49).

The Random Forest (RF) classifier was selected partly due to its strong built-in mechanisms for controlling overfitting and improving model generalization. In fact, each algorithm has its strengths and weaknesses when performing prediction and classification, even on the same dataset (31, 36). Combining classifiers is proposed to enhance the performance of individual classifiers (32) by generating a more precise model for predicting tasks. Such a combination is known as ensemble (65). RF is an ensemble tree-based algorithm that constructs a large number of decision trees using bootstrap aggregation (bagging) and random subspace feature selection. Each tree is trained on a different bootstrap sample of the training data while considering only a random subset of features at each split. This dual randomness reduces the correlation between trees, lowers model variance, and limits the tendency of individual trees to overfit the training data. The final class prediction is produced through majority voting across all trees, which further stabilizes predictions and improves robustness (13).

In addition to the algorithm's inherent overfitting resistance, model validation procedures were applied during experimentation to ensure generalization performance rather than memorization of the training set. Ensemble learning methods, including Random Forest and the proposed voting ensemble, were adopted specifically to address the bias–variance trade-off, which is a central challenge in supervised learning (44). By aggregating predictions from multiple diverse base learners, ensemble models reduce variance and improve generalization compared to single classifiers, thereby mitigating overfitting risk (37, 49).

Random Forest has been widely and successfully applied in bioinformatics and medical prediction tasks—including gene-effect analysis, feature selection, and chronic disease diagnosis — where controlling overfitting and ensuring stable predictive performance are critical requirements (13, 27, 50).

Meta-Vote: is the ensemble algorithm that combines similar or different classifiers through a majority vote (Hard vote) (28) or the average probability (Soft vote) (13) to generate the final prediction results. Recent studies have given greater attention to Meta-Vote for predicting and classifying diseases like diabetes and breast cancer. Therefore, this study would apply both mechanisms to aggregate the prediction results from the heterogeneous classifiers to identify the best strategy based on the dataset collected from the SQUH. The top five classification algorithms were applied to build the voting models, including KNN, NB, SVM, RF and J48(C4.5).

The parameters for constructing the ensemble models were the default applied in the WEKA tool. The DT (C4.5) consists of 0.25 “ConfidenceFactor” for pruning, 2 minimum number of objects in the leaf node and 3 number of the fold to determine the amount of data used for “reduced-error pruning”. In the SVM algorithm, the study utilised “PolyKernel” with exponent=1 and the value of C = 1, which controls the flexibility of drawing the margin to separate between the TRUE and FALSE classes (51). A hundred trees were applied to build RF algorithms. In KNN, the number of the nearest neighbours was k = 1; finally, the NB classifier is non-parametric. Two trials were conducted to build each voting algorithm to identify the best combination strategies based on the dataset collected from the SQUH. The first trial was with the imbalance dataset and the second experiment was with the balanced dataset.

## 4 Material and methods

This study was carried out in strict accordance with the recommendations of the Information System Department at the College of Economics and Political Sciences. The data related to the patients was obtained from a large government hospital. This article does not contain any studies with human participants or animals performed by any authors. All experiments and efforts were based on the obtained data to improve the diabetes diagnosis system.

The KDD process mode was adopted to conduct the study for the following reasons. The aim of the model was developed without referring to any specific data mining technique or any direct link to a particular enterprise (15, 52). The data are the primary and most permanent assets of the model, and it supports simple and complex data mining projects with the iterative and interactive process of knowledge generation. Furthermore, it consists of clearly defined activities and tasks. Data can be integrated and transformed, evaluation measures can be improved, and the mining process can be refined to achieve the desired project goals with different and more appropriate results (15, 53). It is a complete process model consisting of nine stages.

**Figure 2:**
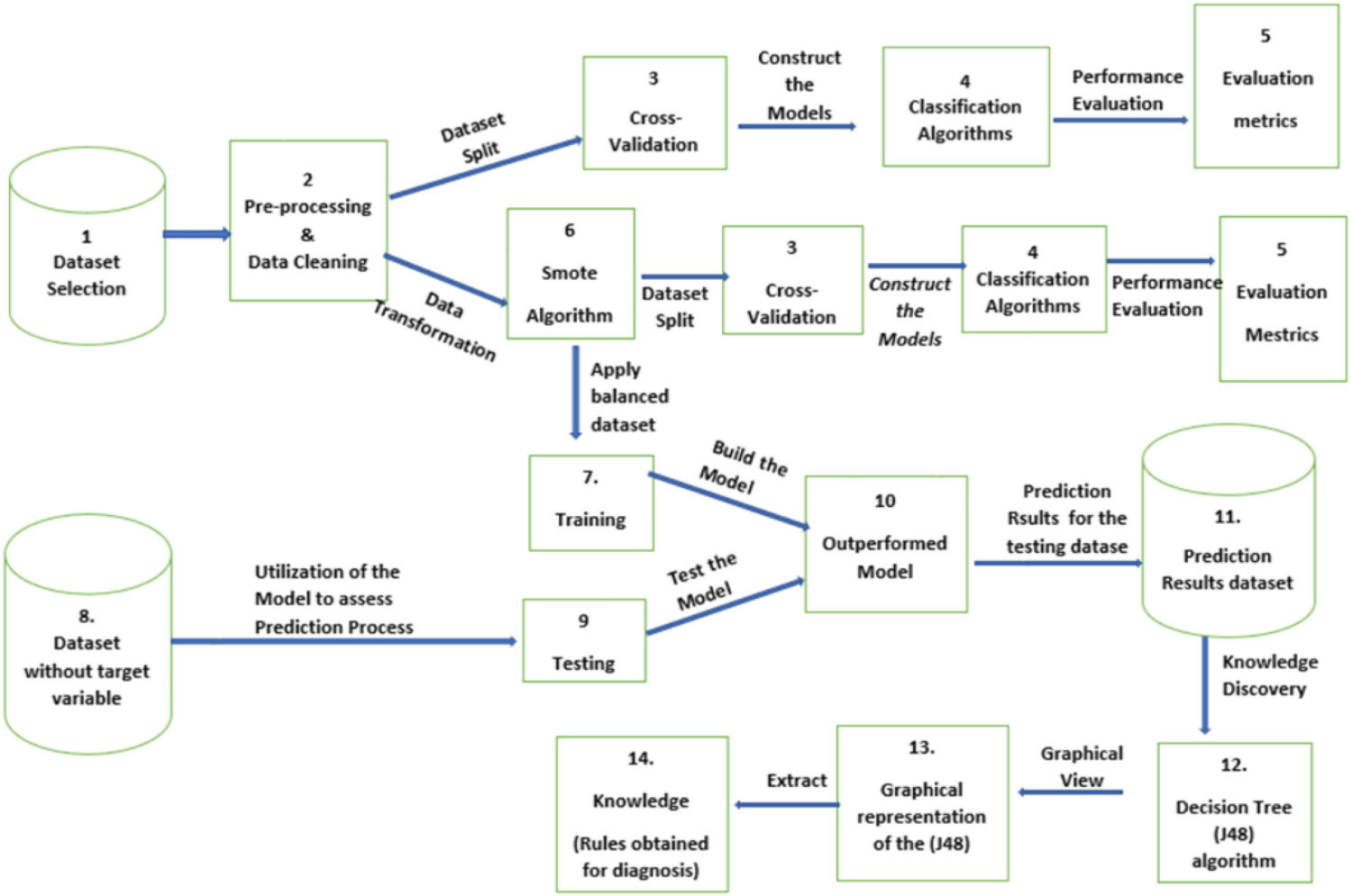
The experiment design to conduct research using the KDD process model.

Figure 2 illustrates the design of the experiments to conduct this research, which follows the KDD process model. To summarize the processes after understanding the application domain and identifying the KDD goal. Initially (1. Data Selection) is the most critical stage to achieve the research objective by building a predictive model based on the most powerful features from the given historical diabetes dataset. The sample dataset is selected from the SQUH repository consisting of twelve diabetes diagnosis features, including the target variable used to classify the patients as either TRUE or FALSE. The next stage is (2. Pre-processing and data cleaning) to prepare a high-quality and powerful predictive dataset for the mining process. The primary process covered in this stage is to improve the dataset's quality, including replacing missing values, removing outliers and extreme values, and applying the information gain function to identify the significant attributes that could be included in the prediction and classification of diabetes. Then the dataset is split using the (3. Cross-validation) method that divides the dataset into ten (10) folds, nine (9) of them participating in training the predicting models, and one (1) fold is used to validate the classification process of the models. This process is repeated ten times to each fold and calculates the average prediction results to provide a final prediction of each (4. Classification model). Finally, evaluate the models using different (5. Evaluation metrics) to identify the outperformed algorithm between those applied in the experiment. To improve the model's accuracy, the [6. Synthetic Minority Oversampling TEchniques (SMOTE)] was applied to increase the number of diabetes instances to balance the target variable (TRUE, FALSE). The processes 3, 4, and 5, repeated to compare the models in different scenarios to improve the accuracy and reliability of the models. The outcome of this stage is to get the most accurate algorithms recommended for clinical use to predict the patients with either true or false diabetes. The last stage is the exploitation of the model by applying the new dataset that does not consist of a target variable to asses the prediction process of the model that has got highest accuracy on the previous processes. The balanced dataset applied to (7. Train) the model with the same procedure as in process 4 while the (8. Dataset without target variable) applied to (9. Test) the capability of the model to classify the patients either true or false diabetes. The (10. Outperform model) would be used to demonstrate this stage. To gain the benefits from the diabetes dataset stored in the SQUH database, the knowledge discovery process was applied to the (11. Prediction results) produced by the selected model. The [12. decision tree (J48)] algorithm is applied to extracting valuable knowledge from the dataset by generating the (13. graphical presentation) of the tree diagram whereas (14. Clinical rules can be derived. This knowledge can be implemented in the clinic to improve the diagnosis system).

### 4.1 Dataset

The experimental dataset was obtained from the Sultan Qaboos University Hospital (SQUH) database in Excel format and covers patient records collected between January 2019 and October 2021 at the Family and Community Medicine Clinic (FAMCO). The dataset contains 4,104 patient records with 14 attributes, including 13 independent variables and one dependent class label indicating diabetic status (TRUE/FALSE). The attributes comprise demographic variables (patient serial number, gender, age), physical measurements (height and weight), vital signs (systolic and diastolic blood pressure), and laboratory test results, including LDL cholesterol, total cholesterol, random blood glucose (RBG), fasting plasma glucose (FPG), HbA1c, and triglycerides. All predictor variables are numeric except gender and the class label, which are nominal. Statistical characteristics of the features show wide value ranges and variability across clinical measurements; for example, age ranges from 21 to 84 years (mean = 51.0, SD = 13.1), while systolic blood pressure has a mean of 133.6 (SD = 31.2). Some attributes contain missing values, most notably RBG (84%), height (34%), and FPG (31%), whereas several core variables such as age, gender, and the class label have no missing data. The class distribution includes 1,451 diabetic cases (40.7%) and 2,653 non-diabetic cases (59.3%), with 1,669 male and 2,435 female patients. Table 1 summarizes the detailed statistical properties, missing-value ratios, and distinct-value counts for all dataset attributes used in the experiments.

**Table 1:** Description of the initial dataset retrieved from SQUH database.

S/No	Attribute	Type	Min	Max	Mean	Std. Deviation	Missing values	Distinct	Unique (%)
1	Patient Serial No	Numeric	124	1464094	-	-	-	4104	4104 (100%)
2	Gender (M = 1, F = 2)	Numeric	-	-	-	-	0%	2	0%
3	Age	Numeric	21	84	51.002	13.141	0%	64	0%
4	Height	Numeric	1.625	1625	159.965	33.941	1407 (34%)	308	176 (4%)
5	Weight	Numeric	6	1124.4	78.488	28.809	282 (7%)	995	399 (10%)
6	Diastolic BP	Numeric	33	680	72.676	14.581	1 (0%)	79	11 (0%)
7	Systolic BP	Numeric	8	1320	133.613	31.241	1 (0%)	124	18 (0%)
8	LDL Cholesterol	Numeric	0.3	16.7	3.133	1.068	523 (13%)	73	11 (0%)
9	Total Cholesterol	Numeric	1.6	48	5.135	1.361	502 (12%)	80	7 (0%)
10	RBG	Numeric	3.3	33.7	6.493	3.209	3454 (84%)	105	40 (1%)
11	FPG	Numeric	3.2	27.1	6.639	2.456	1273 (31%)	151	45 (1%)
12	HbA1c	Numeric	4.1	17.2	6.405	1.568	393 (10%)	109	26 (1%)
13	Triglycerides	Numeric	0.3	156.4	1.745	3.25	501 (12%)	84	24 (1%)
14	Diabetic Labelled: (TRUE & FALSE)	Nominal	-	-	-	-	0 (0%)	2	0 (100%)

LDL is low-density lipoprotein cholesterol (bad cholesterol); RBG is Random Blood Glucose; FPG is Fasting Plasma Glucose; HbA1c is haemoglobin A1C test; Triglyceride is a type of body fat.

### 4.2 Pre-processing and data cleaning

The dataset collected in SQUH consisted of incomplete attributes, inconsistent data, and noisy or outliers that required significant pre-processing (31). The patient's serial number was considered an irrelevant feature; hence, it was removed as it does not have any meaning in the prediction and classification of diabetes (54). Furthermore, the Null values available to each record were replaced by 0 to convert the data from text to numeric. Gender attribute was coded (M = 1 and F = 2) for males and females, respectively.

Missing Values: When included in the experiment, missing values may hinder the model's predicting accuracy. Therefore, attribute RPG was removed from the experimental dataset due to its substantial number of missing values of 84%, as shown in Table 1. The study applied distinct functions to manage the problem of missing values to maintain the number of instances in the dataset and improve the data quality for quality decision-making (55). The “Numeric cleaner” function was used to identify the number of missing values, and “ReplaceMissngWithUserConstant” was applied to five attributes to replace missing values by selecting a particular value that frequently appeared in the distribution due to the availability of the extreme values. These attributes include Height, Weight, FPG, HbA1c, and Triglycerides. The “ReplaceMissingValue” function was another method applied to four features, including Diastolic BP, Systolic BP, LDL Cholesterol, and Total Cholesterol, to replace all missing numeric values using the mean value.

Outliers and Extreme Values were identified using the “InterquartileRange” (IQR) function available in WEKA (43). The result shows that the dataset consists of 355 outliers and 240 extreme values. The “RemovewithValues” function was then applied to remove the dataset's available outliers and extreme values. Thus, the number of cases was reduced to 3,537 with 12 features, among which 1,088 patients have diabetes, and 2,485 are not.

**Figure 3:**
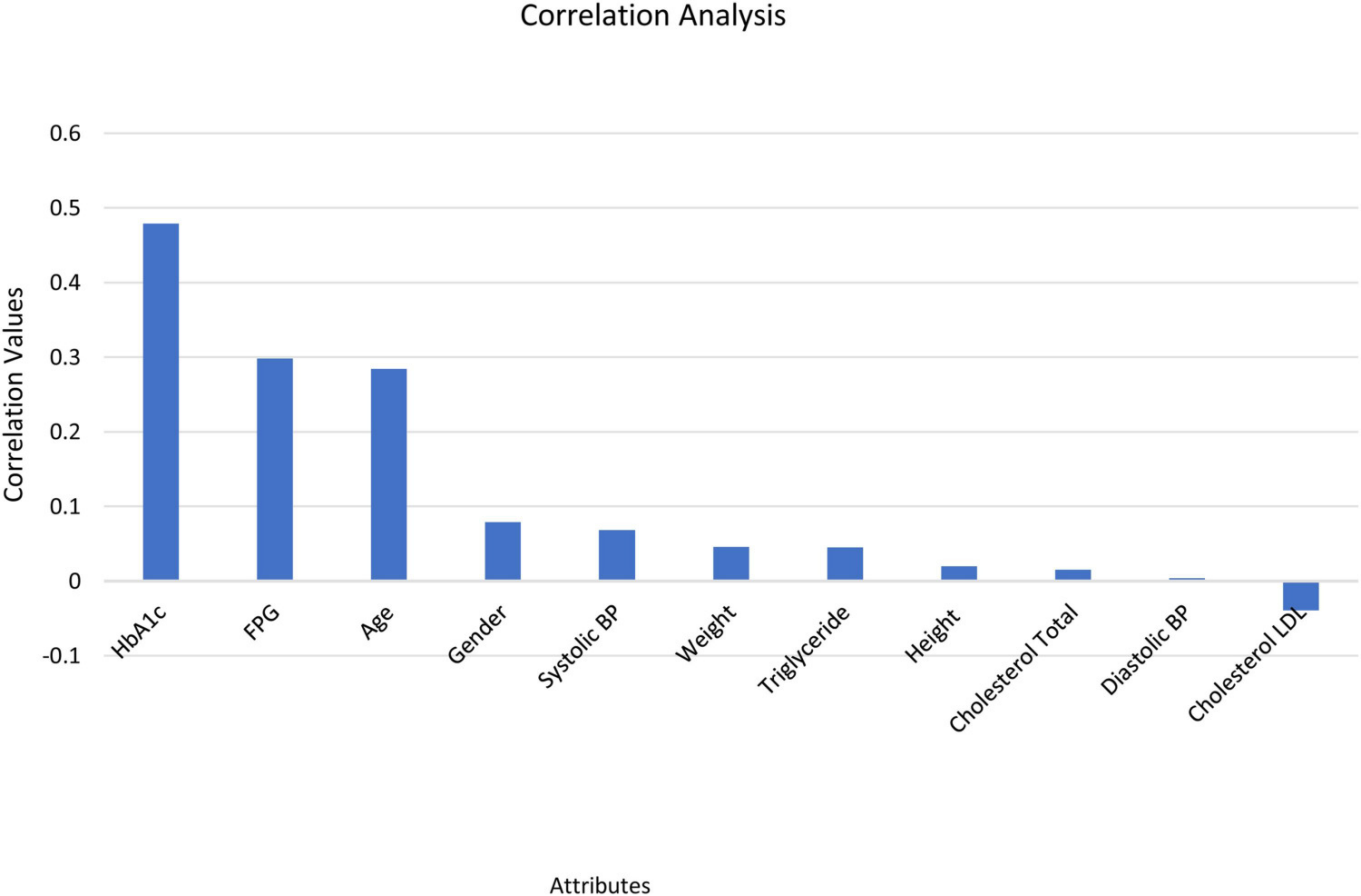
The correlation analysis between the input variables and the target class.

Correlation Analysis was conducted to identify the interrelationship between the independent and dependent variables (38, 45, 56) using a correlation tool in an Excel sheet. Figure 3 illustrates the correlation analysis of the input and output variables of the SQUH-FAMCO dataset. According to the result, the increased blood glucose measured by the HbA1c has a solidly positive correlation with the output class of 0.474 followed by FPG and Age of 0.298 and 0.284.

**Figure 4:**
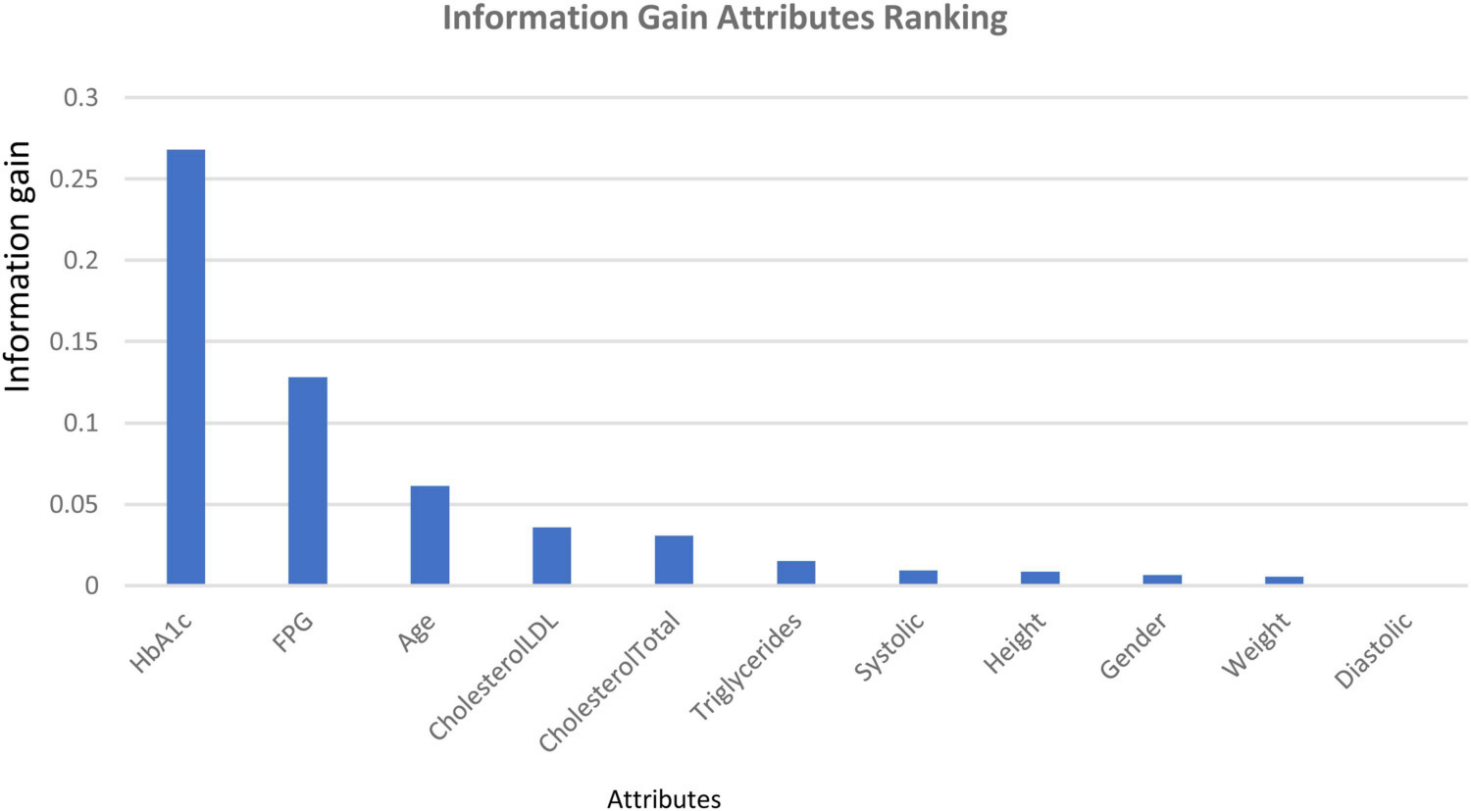
Attributes ranking using information gain with ranker search process.

The feature selection also was conducted using the “InfoGainAtributeEval” function with “Ranker” as a search method. It provides the score for each feature to identify the most relevant features as a final set for the classification process based on the target class (57). Thus, the results show that HbA1c comprises the highest information in the dataset of 0.26793, followed by FPG and Age, as shown in Figure 4 The study applied all features to the experiment, including Gender, Age, height, weight, systolic BP, diastolic BP, LDL cholesterol, Total Cholesterol, HbA1c, FPG, triglycerides, and Diabetic (Diabetic, non-diabetic).

Class Imbalance: The dataset collected from SQUH was imbalanced. It consists of 3,537 instances with twelve attributes, of which 1,088 were diabetic patients and 2,485 were not. When data mining techniques are applied to build the classification models using an imbalanced dataset, the classifier produces high accuracy for the majority classes and less predictive accuracy for the minority class (58). Balancing the output class is necessary for the medical dataset because the minority class represents the disease class (59). To account for the class imbalance problem, the Synthetic Minority Oversampling TEchniques (SMOTE) was applied to increase the diabetic instances that might improve the model's accuracy between TRUE and FALSE classes (60). The minority class increased by a percentage *P* = 125% to each k = 5 nearest neighbour, equal to 1,360 samples. Finally, the total number of instances increased to 4,933, consisting of 2,485 as FALSE and 2,448 as TRUE.

### 4.3 Validation

Cross-validation (CV) is the internal validation process that divides the datasets into K-folds that comprise an equal number of instances to each fold [ (61), p. 116]. Initially, the dataset is divided into K-folds with K-1 subsets, applied to train the models, and one holdout subset is used for validation. Then the model is discarded to build a new model, and the prediction results are maintained. The process is repeated K times to allow each fold to participate once testing the model and K-1 times building the model (62). Then the model's overall performance is calculated using an average score obtained from all iterations. In this research, “Stratified 10-fold cross-validation” was applied; 9 participated in training and 1-fold in testing for 10 iterations. CV provides minimum prediction error results, reduces the model's bias during training (36, 59) and ensures that the class-labelled values are distributed evenly in each stratum (68). Furthermore, the training and testing validation processes are applied to demonstrate the utilisation of the model for the hospital's purpose and to extract valuable knowledge using J48.

### 4.4 Evaluation

The performance of the proposed classification models was evaluated using standard metrics derived from the confusion matrix (61, 63). The confusion matrix provides a structured comparison between the actual class labels and the predicted outcomes and is considered a fundamental tool for assessing classification effectiveness. It summarizes model predictions into four categories: True Positive (TP), True Negative (TN), False Positive (FP), and False Negative (FN), as shown in Table 2.

**Table 2:** Confusion matrix.

Actual value	Predicted Class	
	Positive	Negative
Positive	True Positive (TP)	False Negative (FN)
Negative	False Positive (FP)	True Negative (TN)

While predictive accuracy is commonly used as an overall performance indicator, it may provide misleading conclusions when the class distribution is imbalanced, particularly when the number of negative instances exceeds the positive ones (58, 61). Therefore, additional complementary metrics were used to ensure a balanced and reliable evaluation, including precision, recall (sensitivity), specificity, and false positive rate, see Table 3. These measures provide deeper insight into the model's diagnostic capability, especially for medical decision-support applications where both false negatives and false positives carry clinical risk. The Area under the Roc-curve (AUC) is the graphical visualisation of the model's performance to maximise the True Positive Rate (TPR) and minimise the False Positive Rate(FPR) [ (61), p. 111–113]. AUC represents the binary classifier's ability to correctly classify positive and negative instances. Based on the threshold, the best performance is indicated when the curve lies in the top right corner, where TPR reaches one and FPR reaches zero. In contrast, the worst scenario is to get the curve below the diagonal line between the TPR and FPR.

**Table 3:** Performance evaluation metrics.

Metrics	Mathematical equation	Remarks
Accuracy	$Accuracy=\frac{TP+TN}{TP+TN+FP+FN}$	Total percentage of the correctly classified samples
Precision	$\;\;\;Precision=\frac{TP}{TP+FP}$	The percentage of correctly (positive) classified diabetic samples to the total positive.
Sensitivity or Recall (True Positive Rate)	$\;\;\;\;\;\;Recal=\frac{TP}{TP+FN}$	The percentage of correctly classified diabetic samples over the total diabetic samples
Specificity	$Specificity=\frac{TN}{FP+TN}$	The percentage of correctly classified healthy(non-diabetic) samples over the total non-diabetic samples
1 – Specificity (False Positive Rate)	$\frac{FP}{FP+FN}$	The percentage of incorrectly classified non-diabetic samples over the total incorrectly classified samples
Roc-Curve	Sketched based on the two metrics, true positive rate (TPR) and false-positive rate (FPR), in a simple two-dimensional space that runs between (0 and 1) in both directions.	

## 5 Experimental results

**Figure 5:**
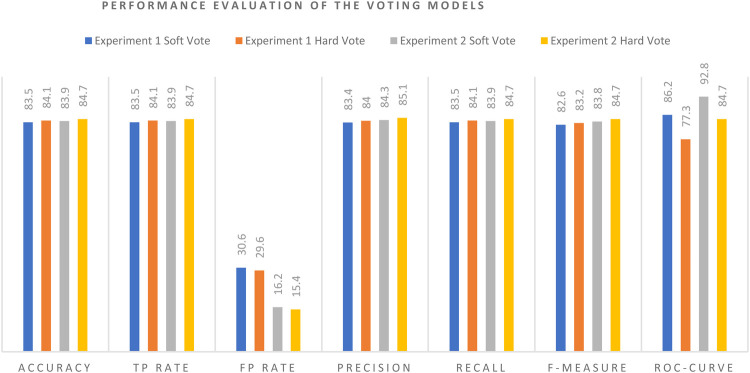
Performance evaluation of the voting models using different combination strategies in experiments 1& 2.

In experiment 1, the number of diabetic patients correctly classified was 651, while the number of non-diabetic cases was 2,353, resulting in an 84.1% predictive accuracy of the hard-vote model, with a total of 3,004 precisely classified cases. The percentage achieved by the AUC curve was 77.3%, which is less significant compared to the soft voting model. Results indicated that the model's correctness consists of an FPR of 29.6%, which is greater weight than the soft voting and a TPR (Sensitivity) of 84.1%. Therefore, the specificity of the model (1 – FPR) is 70.4%. Figure 5 summarises the voting models' performance evaluation based on different combinations of strategies in experiments 1(imbalanced dataset) and experiment 2 (balanced dataset).

The predictive accuracy of the ensemble models is the combination of decisions attained by different individual algorithms (56). In experiment 2, the accuracy of the voting models has improved, as shown in Figure 5. The hard vote achieved the highest accuracy of 84.7% based on the correctly classified instances, of diabetic equal to 1,944 and non-diabetic being 2,235. The average weighted TPR (sensitivity) was 84.7%, and the FPR of 15.4%; the results indicated that the model's specificity was 84.6% (1 – FPR). Thus the number of patients that are non-diabetic classified as a true negative is approximately the same as the diabetic patients classified as true diabetes. However, the AUC curve was 84.7% less than the soft vote. Furthermore, the precision achieved was 85.1%, which specifies the excellent quality of the model that can be used to predict diabetes. Recall and F – measures were 84.7% and 84.7%, respectively. Therefore, the voting model that applies the majority vote mechanism to combine the prediction results from the base classifiers can be used in the clinic to predict patients with TRUE or FALSE diabetes.

**Figure 6:**
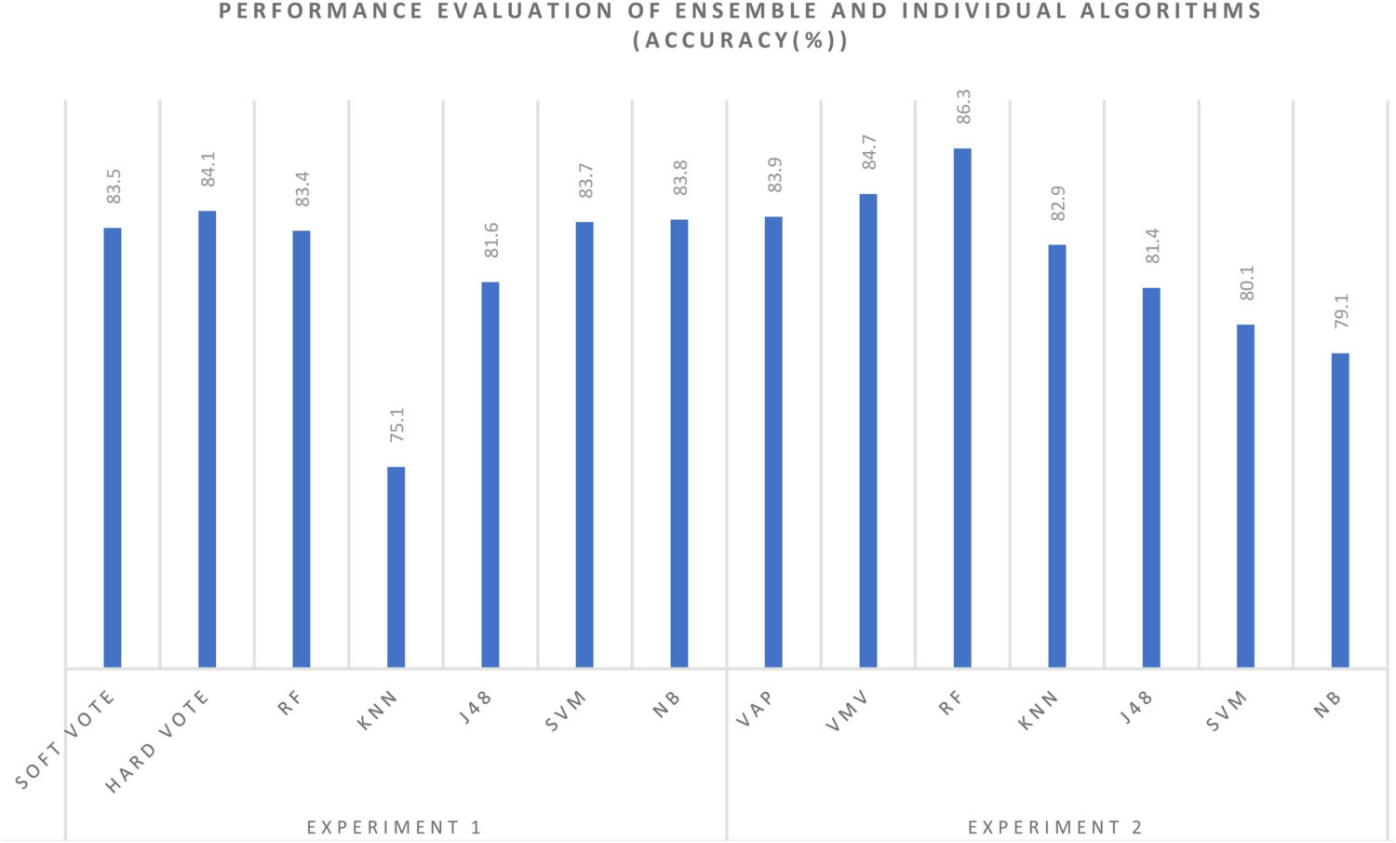
Performance evaluation of voting models with individual algorithms.

A comparison with the individual algorithms using a similar dataset is presented in Figure 6. In experiment 1, the hard vote approaches achieved the highest accuracy of 84.1%, compared to the individual algorithms. The predictive accuracy of the SVM and NB was 83.7% and 83.8%, respectively, which is greater than the soft vote. J48 and KNN achieved 81.6% and 75.9%, respectively.

In experiment 2, the general performance of the models has increased. Nevertheless, the accuracy of J48, SVM, and NB declined to 81.4%, 80.1%, and 79.1%, respectively. The accuracy of the KNN improved from 75.9% to 82.9%. The oversampling techniques provide additional chances for the KNN to predict more accurately. The k-NN algorithm is sensitive to imbalanced datasets since it predicts new instances based on the nearest neighbours (25). These prove that the individual algorithms perform differently based on the circumstances (38), including the number of instances. RF is considered the solid homogeneous ensemble classifier and its contribution improved the performance of the voting ensemble models as it achieved the highest predictive accuracy of 86.3%.

In the KDD process model, the last stage describes the knowledge extraction from the dataset and the exploitation of the model, whereby many of the studies do not provide a detailed illustration in reporting the usefulness of the models for clinical settings (29). The prediction results showed that the hard-vote model achieved the highest reliable accuracy. Therefore, it was selected to demonstrate the utilisation of the model for the hospital settings as it can accurately predict the patients, either diabetic or non-diabetic, by 84.7%.

**Figure 7:**
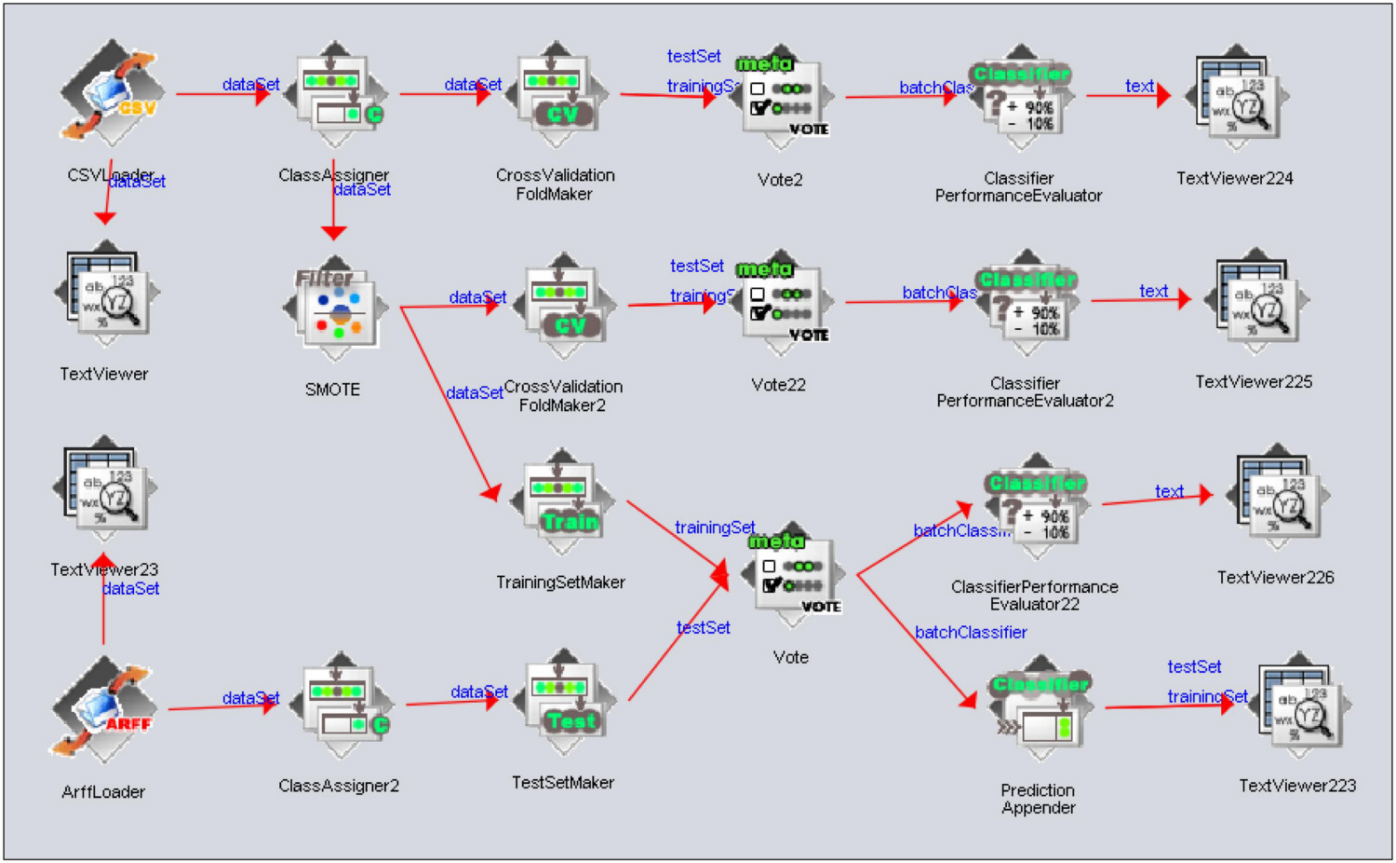
Utilisation of the voting model in predicting diabetes.

The balanced dataset was applied to train the hard-vote model. It consists of 4,933 instances with twelve attributes, 2,485 as FALSE and 2,448 as TRUE. A new file with similar attributes is loaded to predict uncategorised patients and validate the model's utilization for hospital use, as shown in Figure 7. It consists of 3,537 instances with 1,088 patients having diabetes and 2,485 not. The output variable of the new dataset is replaced with “?” to represent the unknown target variable. The prediction results can be viewed and exported to an external file such as a CSV file or text file for further use.

**Figure 8:**
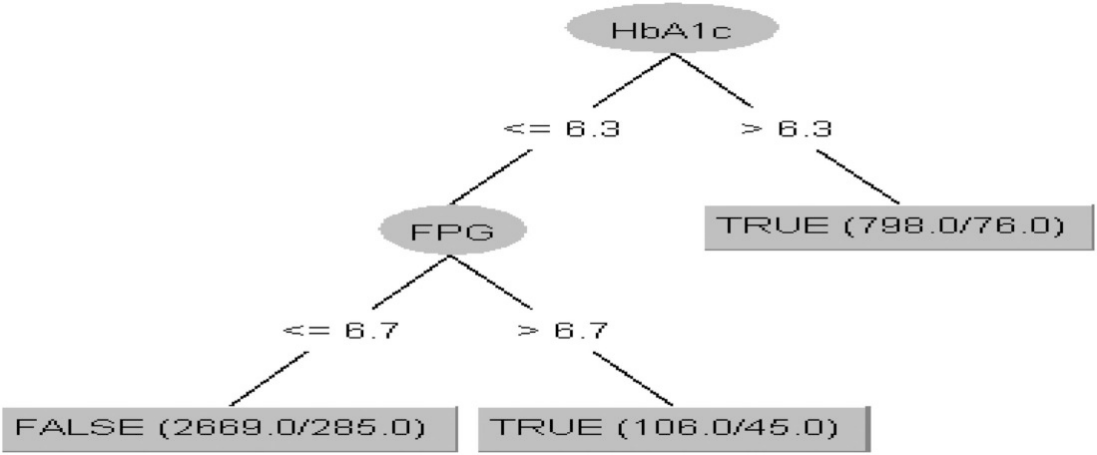
Graphical presentation of the knowledge discovered from the SQUH dataset using J48.

Figure 8 illustrates the graphical presentation of the top three features obtained from the dataset that contribute to the development of diabetes, as shown in Figures 3, 4, including Age, FPG, and HbA1c. J48 was selected to extract valuable knowledge from the dataset (4, 21). The prediction results from experiments 1 and 2 also prove that J48 is a stable algorithm since it consistently produced the approximately same accuracy in both experiments. It performs classification based on the features and the information available to each feature that contributes to the target variable's prediction (31, 32). Table 4 indicates the three rules extracted from the dataset. These rules can be applied during the diagnosis to identify the patient with TRUE or FALSE diabetes. The validity of these statements was determined by the number of patients classified using these rules above one hundred patients,

**Table 4:** Rules extracted from the dataset using J48.

Rule no	Description
Rule 1	If HbA1c > 6.3, then the patient diagnosed = TRUE (798 patients)
Rule 2	If HbA1c <= 6.3 and FPG <= 6.7 then the patient is diagnosed FALSE (2669 patients)
Rule 3	If HbA1c <= 6.3 and FPG > 6.7 then the patient diagnosed TRUE (106patients)

The results show that increases in blood glucose using HbA1c and FPG are considered significant risk factors. This result proves the definition of diabetes discussed in Chapter One (2, 3). Barakat et al. (21) also support this statement by using FPG as one of the rule antecedents in predicting diabetes. Additionally, correlation analysis and Feature selection found that HbA1c, FPG and Age highly contribute to the development of diabetes; however, Age is insignificant in the extracted rules. Similar to Baraka et al. 2010, Age does not appear as a rule antecedent. According to the SQUH dataset, the initial cut-off point of HbA1c extracted from the actual patient's data is 6.3, which shows the variation from the one recommended by the Ministry of Health 2015 using the American Diabetes Association (ADA) criterion. In the truest cases, HbA1c should be greater than 6.3, and in FALSE diabetes cases, the HbA1c should be less than or equal to 6.3, and the FPG should be less than or equal to 6.7, which also deviates from the one that recommended by the Ministry of Health, 2015.

To verify whether the improvement achieved by the hard voting ensemble in Experiment 1 is statistically significant compared with the individual classifiers, McNemar's significance test was applied. This test is appropriate because all models were evaluated on the same test instances and focuses on the disagreement cases between two classifiers. The contingency tables were constructed using the instance-level prediction outcomes of the voting model and each individual classifier (SVM, NB, J48, and KNN). The McNemar test statistic was computed for each pairwise comparison. The results showed that the number of instances correctly classified by the voting model but misclassified by the individual classifiers was consistently higher than the reverse case, yielding *χ*^2^ values above the critical threshold (3.84 at *p* < 0.05). This indicates that the performance gain of the voting ensemble over the individual algorithms in Experiment 1 is statistically significant and not due to random variation.

## 6 Discussion

The results demonstrate that ensemble voting methods, particularly the hard-vote strategy, consistently provide strong predictive performance across both imbalanced and balanced datasets. Performance improvement in Experiment 2 indicates that dataset balancing positively influences classification reliability, especially by reducing false positives and increasing specificity. The ensemble approach outperformed most individual classifiers, confirming that combining multiple model decisions improves robustness and predictive stability. This aligns with prior work showing that ensemble predictions benefit from classifier diversity and decision aggregation (56). Performance variation among individual classifiers across experiments highlights sensitivity to dataset distribution and sample size. The improvement observed in KNN after balancing supports findings that KNN is particularly sensitive to class imbalance due to its neighbour-based decision mechanism (25). Random Forest's strong standalone performance also explains its positive contribution within the voting ensemble.

From a clinical perspective, the hard-vote model's stable accuracy and balanced sensitivity–specificity profile suggest it is suitable for practical deployment in hospital screening scenarios. Unlike many prior studies that stop at model accuracy reporting, this work demonstrates operational model utilisation using unseen patient records, supporting real-world applicability (29). The extracted decision rules further enhance interpretability. HbA1c and FPG emerged as dominant predictors, consistent with established diabetes diagnostic criteria (2). Interestingly, the derived HbA1c threshold (6.3) differs slightly from Ministry of Health and ADA guideline values, suggesting dataset-specific clinical variation. Similar observations were reported by Barakat et al. (21), where Age also did not appear as a rule antecedent despite showing statistical correlation.

Finally, McNemar's test confirms that the ensemble's improvement over individual classifiers is statistically significant rather than due to random variation, strengthening confidence in the ensemble approach.

## 7 External validation of the models

This study compared the previous research that proposed the same voting algorithms using different classification algorithms. Most studies applied the Pima Indian Diabetes Dataset (PIDD), an international dataset retrieved from the UCI machine learning source containing 768 instances with eight input variables and one output variable (Diabetic, nondiabetic).

In the research area of machine learning in classifying and predicting diabetes using the real-patient historical dataset available in the database, none of the previous research applied datasets collected from the SQUH-FAMCO clinic for constructing predictive ensemble models. However, limited research applied traditional data mining techniques using the dataset collected from the National Diabetes Surveys conducted in the Sultanate of Oman. None of the studies was conducted by applying a historical patient dataset collected from the hospital database. This study is the first one in machine learning to classify and predict diabetes using the dataset collected from the SQUH database.

Therefore, compared to recent international studies in classifying and predicting diabetes using voting ensemble algorithms. Five practical classification algorithms were chosen to construct voting meta-algorithms to improve the model's predictive accuracy. The selection is due to the reason that, in the previous studies, they proved to have better prediction results. Hence the models built in this study achieved the highest performance than other recent studies in terms of accuracy and other evaluation metrics. Table 5.1 shows that the models applied in this study achieved better accuracy compared to previous recent studies.

**Table 5:** Comparison of voting models with the previous studies.

Source	Proposed models	Accuracy
Prema et al. (2019)	Hard-Vote, using the majority voting rule, consists of eleven algorithms (KNN, Linear SVM, RBF SVM, LR, DT, NB, RF, Ada Boost, and Gaussian Process and QDA)	Experiment1:80.95% Experiment 2:79.53%.
Kumari et al. (2021)	Soft-Vote using average probabilities consists of three base Classifier (RF, LR, and NB)	79.08%
In this study	Hard-Vote using majority vote consists of five base classifiers (J48, SVM, K-NN, NB, and RF)	Experiment1: **84.1%** Experiment 2: **84.7%**
In this study	Soft-Vote consists of five base classifiers (J48, SVM, K-NN, NB, and RF)	Experiment 1:**83.5%** Experiment 2: **83.9%**

The hard vote achieved the highest accuracy of 84.7% compared to the soft vote obtained 83.9% in predicting diabetes. However, there were some declinations in the predictive accuracy of the base classifiers in the second experiment; both voting models illustrate their ability to combine the prediction results that improve the performance of the models. These models achieved the highest predictive accuracy compared to the recently conducted studies. For example, the study conducted by Prema et al. (28) applied hard-vote using two validation processes. When the dataset was applied to the cross-validation process, the prediction results achieved 80.95%. When the dataset was divided into 70% and 30% training and testing, the model achieved 79.53%. Besides, Kumari et al. (13) proposed a soft vote using the training and testing validation process; the prediction results achieved was 79.04%. Therefore, the hard vote is superior to the soft vote. It depends on the correctly classified instances from the majority base classifiers rather than combining the prediction results by calculating the average probabilities from the individual classifiers.

Finally, as per the KDD processes, these results were presented and discussed with nine doctors from the SQUH, including males and females. They all agreed that increased blood glucose increases the probability of developing diabetes. Age is also a significant risk factor in the experiment results. These criteria are valid as they are also applied to the diagnosis of diabetes based on ADA standards. Besides, the SQUH treats chronic diabetes patients; domain experts suggested that the rules extracted from the dataset might be used for outpatients to control their blood glucose levels. However, this study has a limited number of risk factors that increase the chances of diabetes, such as family relations, BMI, and other environmental factors. The initial diagnosis cut-off point according to the SQUH dataset showed a variation compared to the ADA criteria (6) for HbA1c and FPG of 6.3 and 6.7, respectively. Al Lawati and Barakat (64) also prove this statement by stating that there is no “universal cut-off point” in diagnosing diabetes. The cut-off point varies from one population to the other. Thus, the initial cut-off point should be revised according to the Omani community to reduce the prevalence of diabetes in the Sultanate of Oman.

## 8 Implications

This research addresses the diabetes challenges in the Sultanate of Oman that need to be tackled by improving the diabetes diagnosis system. Beyond merely applying machine learning to a new dataset, the present study advances the field in several methodological, clinical, and validation-oriented dimensions. First, it introduces and systematically evaluates a heterogeneous ensemble voting framework (hard and soft voting) that integrates five diverse classifiers and demonstrates statistically significant performance gains over individual models using McNemar's test, rather than relying only on raw accuracy comparisons. Second, it implements a full Knowledge Discovery in Databases (KDD) pipeline with rigorous real-world clinical data preparation—including missing-value treatment, outlier and extreme-value removal, feature ranking, correlation analysis, and class-imbalance correction using SMOTE—providing a reproducible blueprint for handling noisy hospital datasets. Third, the study goes beyond predictive performance reporting by demonstrating operational model utilization on unseen patient records and by extracting interpretable clinical decision rules (via J48) that were reviewed by domain experts, directly supporting clinical decision-making. Finally, it provides population-specific diagnostic thresholds derived from real hospital data and compares ensemble strategies across balanced and imbalanced scenarios, thereby contributing practical, statistically validated, and clinically interpretable advances rather than a simple dataset-specific model application.

The finding of this study provides evidence that may direct the Ministry of Health decision-makers who aim to reduce the prevalence of diabetes as included in the 2050 Vision, to replan and enhance the screening system with its diagnostic criteria. The execution of correlation analysis and feature selection addresses the most common factors in predicting diabetes, including HbA1c, FPG and Age. These might help doctors and physicians better understand the disease and its interrelationship with other variables to enhance proper treatment.

Furthermore, this research focuses beyond the existing knowledge using KDD and data mining techniques in predicting diabetes by reporting on how the predictive models can be used in the clinical setting. It offers predictive models that can accurately predict the new patient's TRUE or FALSE diabetes at the initial pre-diabetes stage. The prediction results can be viewed, and physician might compare their prediction and the model prediction of diabetes to reduce human error during the decision-making to improve the prognosis of the disease. It offers the extracted rules that doctors can use to control blood glucose, particularly for diabetic chronic patients. Therefore, this approach may reduce hospitalisation and improve healthcare outcomes, reducing health expenditure for both government and individuals.

Moreover, the study contributes to science scholars and bioinformatics by building sophisticated predictive voting models that can predict diabetes patients at the initial pre-diabetes stage using the historical dataset collected from the government hospital. These ensemble models were statistically evaluated using predictive accuracy and other evaluation metrics such as precision, recall, F-Measure and AUC curves to prove the correctness of the models. Besides voting models compared to the five individual models and the previous recent studies that applied similar methods in predicting diabetes. Therefore, this research can help scholars who want to enhance the study using data mining tools and techniques in predicting diabetes and in different industrial domains to predict and classify categorical variables.

## 9 Conclusion

This study presented a robust ensemble voting framework for early-stage diabetes prediction using real clinical data from the SQUH database. A comprehensive knowledge discovery in databases (KDD) pipeline was implemented, encompassing extensive data preprocessing, cleaning, feature preparation, bias mitigation, and multi-model ensemble construction. The raw dataset contained incomplete, inconsistent, and noisy attributes, requiring significant preprocessing—over 80% of the effort—to generate high-quality data for unbiased results. This process included handling missing values, outliers, and extreme values, removing irrelevant features such as serial numbers, converting textual Null values to numeric, balancing the TRUE and FALSE diabetes instances, and performing feature selection and correlation analysis. The analysis identified HbA1c, followed by FPG and Age, as the most significant predictors of diabetes, with HbA1c serving as an initial diagnostic criterion, potentially simplifying patient classification and guiding outpatient blood glucose control. Notably, the cut-off points for HbA1c and FPG derived from the dataset differed from the ADA standards, suggesting that community-specific thresholds in Oman could reduce diabetes prevalence through early intervention.

The ensemble framework integrated five diverse classifiers—J48, KNN, SVM, Random Forest, and Naïve Bayes—using hard and soft voting strategies to improve predictive reliability and overcome the limitations of individual models, such as overfitting and high variance. The hard-voting model achieved the highest accuracy of 84.7% and an AUC of 84.7%, demonstrating strong performance in distinguishing between TRUE and FALSE diabetes cases. Random Forest also exhibited strong individual performance, contributing to overall ensemble robustness. These results highlight that combining diverse base classifiers enhances generalization, reduces variance, and produces stable predictions across both imbalanced and balanced datasets.

Beyond predictive performance, this study provides a methodologically transparent and clinically applicable approach, including reproducible parameter configurations, bias handling, and deployment-oriented validation using unseen patient records. Overall, the proposed ensemble voting framework offers a reliable, extensible, and practical foundation for AI-assisted diabetes prediction in healthcare settings, supporting early diagnosis and personalized patient care.

## Data Availability

The raw data supporting the conclusions of this article are subject to the ethical guidelines and regulations of Sultan Qaboos University. Requests to access the datasets should be directed to the corresponding author/s.
